# EEG Frequency Changes Prior to Making Errors in an Easy Stroop Task

**DOI:** 10.3389/fnhum.2017.00521

**Published:** 2017-10-31

**Authors:** Rachel Atchley, Daniel Klee, Barry Oken

**Affiliations:** Department of Neurology, Oregon Health and Science University, Portland, OR, United States

**Keywords:** mind-wandering, attention, theta, alpha, executive control

## Abstract

**Background:** Mind-wandering is a form of off-task attention that has been associated with negative affect and rumination. The goal of this study was to assess potential electroencephalographic markers of task-unrelated thought, or mind-wandering state, as related to error rates during a specialized cognitive task. We used EEG to record frontal frequency band activity while participants completed a Stroop task that was modified to induce boredom, task-unrelated thought, and therefore mind-wandering.

**Methods:** A convenience sample of 27 older adults (50–80 years) completed a computerized Stroop matching task. Half of the Stroop trials were congruent (word/color match), and the other half were incongruent (mismatched). Behavioral data and EEG recordings were assessed. EEG analysis focused on the 1-s epochs prior to stimulus presentation in order to compare trials followed by correct versus incorrect responses.

**Results:** Participants made errors on 9% of incongruent trials. There were no errors on congruent trials. There was a decrease in alpha and theta band activity during the epochs followed by error responses.

**Conclusion:** Although replication of these results is necessary, these findings suggest that potential mind-wandering, as evidenced by errors, can be characterized by a decrease in alpha and theta activity compared to on-task, accurate performance periods.

## Introduction

Mind-wandering is a form of off-task attention that has been associated with negative affect and rumination (Scheeringa et al., 2008; Lagopoulos et al., 2009; Hasenkamp et al., 2012; Sood and Jones, 2013; Levinson et al., 2014; Wilson et al., 2014; Smallwood and Schooler, 2015). Mind-wandering can be an adaptive and even a natural state, but it offers potential drawbacks as well. Smallwood and Schooler (2006) hypothesized that mind-wandering is associated with executive control and that mind-wandering precipitates attentional shifts that can impair task performance and awareness of external sensory information. Certain inflexible and negative forms of mind-wandering, such as perseverative cognition, can have deleterious effects on the health and mood of persons with Major Depressive Disorder (Ottaviani et al., 2015), for example.

Previous studies have assessed mind-wandering using objective brain activity measures such as attentional P3 event-related potential (ERP) markers in conjunction with behavioral and self-report measures (Smallwood et al., 2008), fMRI for default-mode network assessment and self-report (Mason et al., 2007), and fMRI for brain region activity levels during mind-wandering (Weissman et al., 2006). It was found that mind-wandering naturally occurs as sustained attention waxes and wanes (Smallwood et al., 2008), that default mode network recruitment was greater during periods of higher self-reported mind-wandering (Mason et al., 2007) and that the start of mind-wandering events can be traced to pre-stimulus brain activity in the right prefrontal regions and the anterior cingulate (Weissman et al., 2006).

Additional ERP studies on the topic of mind-wandering have found that the amplitude of the P1 component, as an early occurring ERP that occurs in response to stimuli, was decreased when participants reported they were engaged in task-unrelated thought (Baird et al., 2014). Furthermore, the Baird et al. (2014) study conducted a time-frequency analysis and observed a decrease in theta-band cortical phase-locking over the parietal areas, as well as increased cortical processing during task-relevant action. The authors interpreted these findings as evidence that there is an increase in neural processing in an effort to recouple attention following a mind-wandering event.

Recent research by Baldwin et al. (2017) explored the effects of mind-wandering on behavioral responses. Participants who completed various simulated driving tasks were asked to self-report mind-wandering and were also intermittently prompted to report whether they were experiencing mind-wandering. While mind-wandering events affected performance on the driving task in terms of reduced driver speed and less lane variability, the electrophysiological findings of this study included an increase in alpha-band power during mind-wandering and a reduced amplitude in the P3a component during auditory probes.

Event-related potential studies of mind-wandering show that attentional ERP components such as P3 are reduced during task-unrelated thought. For example, Kam et al. (2012) also studied the effects of mind-wandering on behavior and found that the P3 attentional component was reduced when participants self-reported mind-wandering during a motor tracking task, and that errors on the task were also increased. These results support the findings of the study Baldwin et al. (2017) and suggest that mind-wandering can have functional effects on motor control and behavior. O’Connell and colleagues found that mind-wandering, as lapsing attention, was characterized by an increase in alpha band activity up to 20 s before errors were made on a task in which the goal was to identify a rare-occurring visual target. After a missed target, the authors observed reductions in the amplitude of the P3 component as well.

We wanted to build on this research by assessing mind-wandering prior to errors, and to develop a covert and objective assessment of mind-wandering state without affecting behavior or introducing the subjectivity of self-report. We used electroencephalography to record frontal frequency band activity while participants completed a computerized Stroop task. The task was designed to require constant attention in order to avoid errors, while being uninteresting enough to increase the likelihood of one aspect of mind-wandering, task-unrelated thought (Christoff et al., 2016). More specifically, the mind-wandering task in this experiment was designed in such a way that it was easy for participants to maintain high levels of accuracy while facing no significant time pressure for a response. Thus, all errors, including those potentially related to category response inhibition failure, were likely mind-wandering errors. Decreased theta activity has been particularly related to worse cognitive performance including working memory (Onton et al., 2005; Sauseng et al., 2007; Nigbur et al., 2011). Decreased alpha can be intuited as a characteristic of mind-wandering when drawing from previous work in which increased attention, specifically meditation, showed increased alpha (Lagopoulos et al., 2009; Ahani et al., 2013, 2014). Although there are several potential contributors to errors on a task potentially related to mind-wandering, we hypothesized that mind-wandering would be characterized by decreases in theta and alpha frequencies across the frontal regions of the brain.

## Materials and Methods

### Participants

Twenty-seven older adults (*M* age = 60 years, *SD* = 6) were enrolled in a mindfulness meditation randomized controlled trial. The sample was 81% female and well-educated, with an average of 16 years of schooling. To meet inclusion criteria, participants needed to be generally healthy, at least mildly stress with a Perceived Stress Scale Score (PSS) (Cohen and Williamson, 1988) ≥9, and without current medical illness or untreated depression. Older adult participants were used for this study as a convenience sample since they were participating in a still-ongoing intervention study within our laboratory. All data presented here were acquired at baseline, prior to any intervention.

### Study Design

Participants completed a computerized Stroop matching task before being randomized to mindfulness training, education control, or wait list control groups in a larger intervention study. Group findings will be discussed in a future intervention paper when the study is complete.

### Stimuli

**FIGURE 1:**
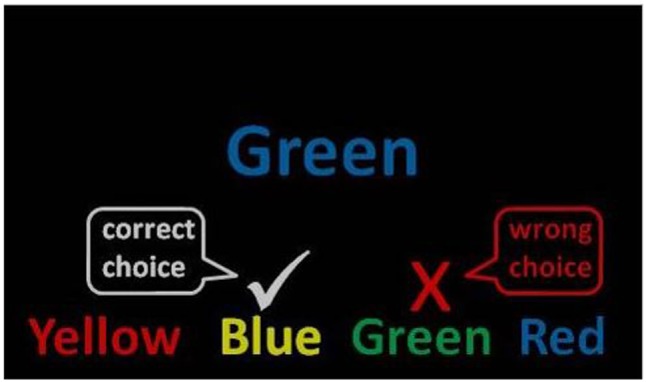
Mind-wandering Stroop task. Example of an incongruent (mismatched) trial used in the instructions. A congruent trial would present the word green in the color green.

The stimuli were the words red, yellow, blue, and green presented in Eprime 2.0 using the standard colors for red, yellow, blue, and green (Schneider et al., 2002). One target item was presented in the center of the computer screen with four answer options beneath. The stimuli were presented in large, readable Arial font and remained on the screen until an answer was chosen or a timeout occurred (see **Figure 1**). Word color and word text were counterbalanced.

### Apparatus

The mind-wandering task was built and administered in Eprime 2.0 and presented to participants on a 40 cm × 30 cm CRT monitor with a refresh rate of 60 Hz. Viewing distance was 90 cm. Participants logged responses on keyboard, using the numbers 1–4 on the number pad.

### Procedure

Participants completed the task in a quiet room with adequate lighting to see a keyboard. They used the keys 1–4 to register their responses. The instructions were presented on a computer screen in front of the participant: “Below is an example of what the next task will look like on the screen (**Figure 1**). Notice that the words correspond to four different colors (red, green, blue, and yellow). Also notice that each word is written in a different color ink. To succeed in this task, you will need to match the color ink of the target word presented in the center of the screen with one of the typed words presented at the bottom (ignoring the color in which it is written). You should be as accurate and fast as possible.” Importantly, accuracy was stressed over speed by the experimenter showing participants how to do the task.

Next, there were two practice tasks: one with 16 congruent trials and one with 16 incongruent trials. Participants could repeat the practice tasks until they understood the controls and directions. Half of the Stroop trials were congruent (word/color match), and the other half were incongruent (mismatched). Participants completed 288 trials (*SD* = 11.7) within the 15-min testing period. The jitter timing (3–7 s stimulus presentation intervals) and switching between congruent/incongruent conditions contributed to the modified challenge and novelty of the task in capturing mind-wandering state.

The relatively slow stimulus presentation rate was chosen to facilitate some degree of boredom and subsequent mind-wandering. Behavioral response data were collected in addition to EEG recordings. Error rates were defined as the percentage of incorrect responses in comparison to the number of correct responses. Impulse or inhibitory failure errors were ruled out as responses that occurred less than a second after stimulus presentation and we not included in analyses, but this occurred only a handful of times across the entire data set. Otherwise, reaction time was not a concern in this study beyond time-out errors, which were not included in the analyses. The task took approximately 15 min to complete including practice.

### EEG Data

EEG was recorded with Ag-AgCl passive-sensor electrodes connected to a BioSemi 32-channell, 24-bit resolution, ActiveTwo system (BioSemi, Amsterdam, the Netherlands). All data were sampled at 1024 Hz with amplification bandwidth settings at 0.1–70.0 Hz. EEG data from frontal electrodes (Fz, FC1, FC2, F3, F4) were assessed in BrainVision Analyzer (Version 2.1.1.327, Professional Edition) with offline filters set at 70 Hz low pass, 0.1 Hz high pass, and 60 notch. Artifact rejection was semi-automated to remove instances of signal contamination due to electrical interference or muscle movement, as described in Wahbeh and Oken (2013). Eye-blink artifact was minimized using BrainVision’s semi-automatic Independent Component Analyses (ICA) feature and the InfoMax (Gradient) Restricted algorithm, with visual inspection and confirmation of ocular corrections. Raw theta and alpha amplitudes were used in the analyses.

Frequency spectra ranges were defined as 8–15 Hz for alpha activity and 4–7 Hz for theta activity. Responses were automatically coded as correct or incorrect from Eprime to BrainVision. One-second epochs prior to stimulus presentation were used to compare trials followed by correct versus incorrect responses. One second was the minimum amount of time needed to obtain enough EEG data, while any more than that would have run into ERPs from the previous trial. Post-stimulus activity was not examined in this paper since feedback-related potentials, such as long-latency feedback-related negativity, could interfere with the mind-wandering measure. We also intended to capture mind-wandering covertly as it happened, prior to stimulus processing and motor response.

### Sleep Analysis

All EEG recordings were assessed for signs of drowsiness, defined as entering stage 1 sleep (Iber et al., 2007) in intervals that overlap with pre-stimulus activity. Recordings were analyzed by two authors trained in sleep scoring.

### Statistical Analysis

Error counts, error rates (number of error responses divided by the total number of responses), and average alpha and theta power across all frontal electrodes were analyzed in paired-samples *t*-tests. Statistical analyses were conducted in SPSS (Version 22, IBM). The significance level used was *p* < 0.05.

## Results

### Task Performance

Participants made errors on the incongruent trials, with an overall average error count of *M* = 22.65*, SD* = 22.35, and an overall error rate of *M* = 9.4%*, SD* = 0.100. Errors on congruent trials did not occur outside practice sessions, which were not analyzed. Pearson correlations were run for all EEG and behavioral variables in regard to age and gender factors, but no significant relationships were present.

### EEG Findings

Five participants’ EEG data were excluded. Two participants had excessive artifact because they were unable to stay sitting still. Another participant made no errors on the Stroop task. Two participants were unable to understand the incongruent task instructions despite repeated practice trials.

**FIGURE 2:**
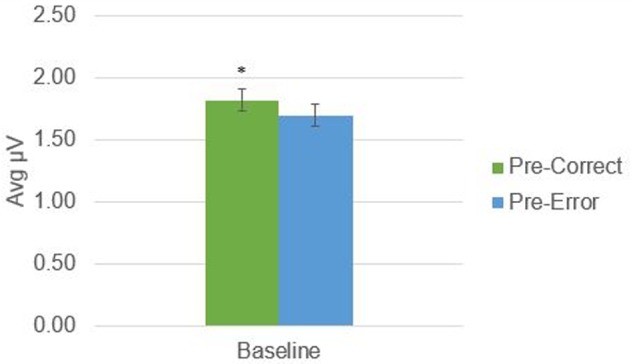
Alpha activity in 1-s epochs preceding stimulus presentations. ^∗^Indicates significant difference at *p* < 0.05.

**FIGURE 3:**
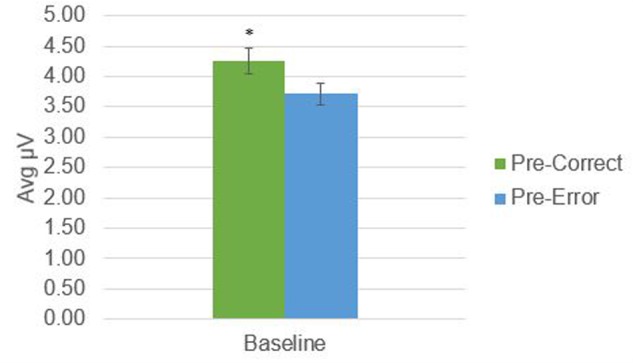
Theta activity in 1-s epochs preceding stimulus presentations. ^∗^Indicates significant difference at *p* = 0.015.

There was a significant difference in alpha amplitude between correct and error trials (*M* = 0.11 μV*, SD* = 0.26), *t*(21) = 2.09, *p* = 0.049, as well as theta amplitude (*M* = 0.54 μV*, SD* = 0.95), *t*(21) = 2.65, *p* = 0.015 (**Figures 2, 3**). Using conventional EEG scoring, there were no differences in relative number of drowsy epochs among those with higher error rates when compared to those with lower error rates.

## Discussion

Our hypothesis was supported; mind-wandering events evidenced by errors were characterized by decreases in alpha and theta band activity prior to stimulus presentations associated with errors when compared to EEG prior to correct response trials. However, it is difficult to measure mind-wandering directly and subjective reports were not collected. These data must be interpreted with appropriate caution, as task-unrelated thoughts are intertwined with other concepts such as boredom, fatigue, and drowsiness. Our analyses suggested that simple drowsiness was not an obvious cause of the EEG changes prior to errors. The connection between error rates on the new task and mind-wandering is inferred in an effort to capture mind-wandering in a covert manner that will not affect participants’ behavior or emotional state. The connection between behavioral data and task-unrelated thoughts or mind-wandering was carefully crafted by making the modified Stroop task slow and easy enough that there should not have been difficulty errors, including those related to inhibitory failure. The Stroop task is inherently concerned with the conflict between automatic processing and controlled responses, which appeals to the attentional component of task-related thought. The Stroop task was chosen as a basis for this experiment because of this feature. That is, when responses were no longer under considerable timed response pressure, any errors made after the impulse stage of approximately 1 s are likely to be the result of a failure in participant attentional focus.

Participants were usually awake and alert, meaning that the mind-wandering effects are unlikely to be related to sleepiness. Previous work showed that an increase in theta band power was associated with decreased performance associated with drowsiness performance (Makeig and Jung, 1996), while we found the opposite in participants who may have experienced mind-wandering before they made errors. These authors found that a decrease in alpha was also associated with poor performance related to sleep transition. While we observed a decrease in alpha band power, we scored participants’ EEGs for drowsiness and did not observe drowsiness. This signifies an interesting possible relationship between sleep transitions and mind-wandering with differential effects on theta and alpha power.

Moreover, previous research (Gevins et al., 1997) has shown a positive correlation between frontal midline theta and working memory task load, while alpha activity decreased. Interestingly, both alpha and theta activity increased as participants became more practiced in the working memory task and performance improved, regardless of stimulus modality. These results imply that in our experiment, a decrease in alpha may actually indicate processing of a kind that is task-irrelevant as the decrease occurred prior to errors.

However, out results do not match some previous reports on mind-wandering and frequency band activity. We will discuss those results and present some potential explanations. Baldwin et al. (2017) observed an increase in alpha activity with subjective reports of mind-wandering, but this was over parietal regions as they had no results for the alpha band in the frontal areas. Likewise, O’Connell et al. (2009) saw an increase in alpha activity in the parietal region most prominently. The results of this study focused on frontal areas with the idea of gathering information on neural processes related to inhibition and executive control more so than the visual processing focus of these studies.

Other studies have shown findings in line with ours, such as Barron et al. (2011) and Baird et al. (2014) who observed a decrease in theta activity associated with mind-wandering events. Likewise, Gevins et al. (1997) observed that frontal midline theta increased with working memory load demands. Kam et al. (2012) observed an increase in errors associated with mind-wandering, as our data also show. Broader studies on the nature of frequency band activity also describe increases in theta, specifically frontal midline theta, as being associated with increased mental activity (Inanaga, 1998), which could potentially align with the processing demands of task-related thoughts and focused mental effort. Subjective reports of mind-wandering are associated with increases in theta and delta activity, while alpha and beta activity decrease (Braboszcz and Delorme, 2011). Our data are similar to findings by Braboszcz and Delorme (2011) but have an important quantitative difference: in an effort to be more objective, mind-wandering episodes were inferred from behavioral errors rather than self-report. This difference in design may explain why we see a decrease in theta activity. Other have also shown decreased frontal theta is associated with worse cognitive performance, including working memory performance (Onton et al., 2005; Sauseng et al., 2007; Nigbur et al., 2011), an aspect of cognition required for the task in this study. People can press a button to indicate when their mind wanders, or when they lose track of what they are doing, but both of these measures often occur seconds after the fact. A behavioral measure of mind-wandering that does not rely on self-report will be more closely time-locked to discrete mind-wandering events. The mind-wandering task used in this study was built to be easy enough that errors were almost always related to mind-wandering.

In terms of mind-wandering as a broader theoretical concept, Barron et al. (2011) conducted an experiment to better understand the potential cognitive contributors to mind-wandering events. They found that task-unrelated thought occurred in conjunction with reduced cortical processing of events that were task-relevant as well as stimuli intended to be distractions. The authors’ conclusion was that their results supported the decoupling hypothesis of mind-wandering, which is the idea that sensory processing is reduced regardless of task-relevance during mind-wandering. In contrast to mind-wandering, mindfulness meditation is an established complementary medicine technique (Beauchamp-Turner and Levenson, 1992; Kabat-Zinn et al., 1992; Miller et al., 1995; Teasdale et al., 1995, 2000; Grossman et al., 2004; Chiesa and Serretti, 2010; Zeidan et al., 2010, 2012), in which there is a focus on non-judgmental attention regulation. Increased competence in the mindfulness technique can be captured in brain activity (Cahn and Polich, 2006; Moore et al., 2012) and meditators demonstrated increased attentional control (Atchley et al., 2016). The unique pattern observed in this experiment neatly complements previous findings (Lagopoulos et al., 2009; Ahani et al., 2013, 2014) that show an inverse, increased pattern in alpha and theta activity during meditation.

Mind-wandering may present a contrasting state to mindfulness, although the relationship between the two states can be complex, interconnected, and involve switching back and forth (Vago and Zeidan, 2016). Mindfulness meditation training programs have been shown to reduce mind-wandering (Rahl et al., 2016). Training in the acceptance aspect of mindfulness may be involved in improving behavioral aspects of mind-wandering, although the cognitive mechanisms driving this effect are not fully known (Rahl et al., 2016). This provides a promising avenue for future research.

As pointed out by Christoff et al. (2009) in an fMRI analysis of mind-wandering, it is essential for future research to use objective neurophysiological measures and subjective participant reports in tandem in order to maximize successful assessment of mind-wandering state.

## Conclusion

Our results suggest mind-wandering associated with errors on an easy task can be characterized by decreased alpha and theta activity prior to stimulus presentation when compared to EEG prior to stimuli generating a correct response. The modified computerized Stroop task we designed to induce mind-wandering also appears to have been effective for this purpose, although replication of these results is necessary.

## Ethics Statement

This study was carried out in accordance with the recommendations of the Oregon Health & Science University Institutional Review Board with written informed consent from all subjects.

## Author Contributions

RA: Contributed to design, data collection, analysis of results, and writing the manuscript. DK: Contributed to data collection, review of manuscript. BO: Contributed to design, review of manuscript.

## Conflict of Interest Statement

The authors declare that the research was conducted in the absence of any commercial or financial relationships that could be construed as a potential conflict of interest.
